# Progress in Surgery

**Published:** 1903-08-29

**Authors:** 


### Progress in Surgery.

(*Concluded from page* 363.)

### GENITO-URINARY.

**Moveable Kidney.**—Giuseppe Ruggi[3] advocates a new method of fixing the kidney by operation. He maintains that the following principles should be adhered to in these operations :—1. The displaced kidney must be brought to a normal height and position. 2. It must be fixed in such a position that it will receive the normal protection of the thorax. 3. The kidney must not be injured nor its secreting tissue damaged by the operation. 4. Aseptic healing of the wound must be secured. 5. The kidney must be so fixed in its allotted place that its nerves and vessels are exposed to no pressure or dragging. The author believes his operation fulfils these conditions. The special points of the operation are the following :—The lumbo-cutal incision extends from the eleventh rib to slightly beyond the iliac crest, in order to gain plenty of room. The fatty capsule of the kidney is removed as much as possible and all hæmorrhage carefully arrested. The fibrous-capsule is incised on the convex border of the kidney and peeled off on one or both surfaces of the organ. The author considers that he has proved, both by trial in actual practice and on the cadaver, that the capsule must then be artificially prepared in order to withstand the traction to which it is exposed, and for this purpose he rolls the denuded portions in the vertical direction to form a pedicle on each surface. The pedicle is ligatured and transfixed. The capsule is only stripped half way to the hilum of the kidney, and the two poles of the organ prevent the capsule peeling off further. The ends of the ligatures, left long for the purpose, are then passed through the eleventh intercostal space and tied there. In this way the kidney is well drawn up. Finally to support the lower pole of the organ, the so-called fascia of Zuckerkandl is sutured beneath it to the deep surface of the posterior edge of the wound. W. Gill Wylie[4] has also discussed moveable kidney. Many women, he pointed out, presented with this affection a relaxed and congested condition of the generative organs. Also in many cases there was a diseased appendix present and the pain and dragging might be relieved by removing the appendix without replacing the kidney. The writer does not think that surgeons should endeavour actually to fix the kidney, because normally the kidney was somewhat moveable. His object was to suspend the kidney in proper position, and in such a way that it would move slightly with respiration. For this purpose, after replacing the kidney, he pulled out the fatty fibrous capsule with forceps until the tension held the kidney just above the twelfth rib, then cut off the superfluous part and sutured the ends to the fascial lining of the muscles. T. E. Gordon discusses moveable kidney.[5] He has operated on 12 cases by Morris's method with one modification. He states that a certain degree of return of mobility is not inconsistent with perfect success as regards the relief of symptoms. He groups cases of moveable kidney into four clinical classes as follows :—(1) Those with slight discomfort associated with general ill-health, with or without hysterial manifestations ; (2) those with gastro-intestinal symptoms ; (3) those with hepatic symptoms ; and (4) those having distinctly renal symptoms. In one case the writer thought a moveable tumour on the left side was the kidney, but it proved to be a cancer of the stomach. There may be actual dilatation of the stomach as an effect of the dragging of a moveable kidney. The symptoms of moveable kidney sometimes exactly simulate those due to gall-stones. On the other hand, gall-stones and moveable kidney often exist together.

**Congenital Defects of the Kidneys.**— G. J. Winter[6] has contributed a paper on this subject in connection with a case in which a patient with one kidney only was operated upon for a cyst in the same, and quickly died. He groups the defects into three classes—viz., (1) that in which there is no trace of a second kidney ; (2) that in which the two kidneys are fixed together ; and (3) that in which both kidneys were originally present, but in which one has degenerated and atrophied. Winter only considers the first group in detail. In 237 published examples of this class of defect the left kidney was absent 129 times, the right 98 times, and the defect was twice as common in men as in women. The defect was usually discovered between the ages of 20 and 25. An individual with one kidney can live to old age. On the side in which the kidney is absent there are usually no renal blood-vessels, and in almost all the ureter is absent. Only in 18 cases was the latter present, and was then usually very thin and only permeable with a sound for a few centimetres from the vesical end. In one group of cases there was absence of half of the trigone, and in five cases the single ureter opened in the middle line of the bladder. Usually the single kidney present is hypertrophied, and the vessels and ureter larger than normal. In 63 cases the single kidney was diseased. In 25 per cent. of cases the supra-renal gland was absent. Very frequently there were co-existent defects in the genital organs on the side on which the kidney was absent. This was mostly the case in females, and the commonest defect was the existence of a uterus unicornis, but in some cases a uterus bifidus or didelphis or duplex was present. There was a double vagina in two cases, and absence of a vagina in two others. The testicle was completely absent in only four cases, but in other cases it was smaller than normal and atrophic. In 8 per cent. of solitary kidneys calculus was present, either in the kidney or ureter. Operations on solitary kidneys have been sometimes successful, but must be undertaken with great caution. In diagnosis special attention must be given to the state of the genital organs. Palpation is only of use if both kidneys can be felt. Cystoscopy and catheterisation of the ureters yield the most definite evidence of the anomaly.

**Stone in the Ureter.**—Jacobs, of Brussels,[7] has recorded a successful case of transperitoneal ureteral lithotomy for this condition, in which a stone weighing an ounce and a half, and which had probably occupied the ureter for over 30 years was, removed. The patient, a woman of 44, complained of severe attacks of pain referred to the left side of the pelvis, to which she had been liable since 10 years of age. A hard mass could be felt, not tender on pressure, high up on the left side of the pelvis. After each attack of pain pus appeared in the urine. Abscess of the ovary communicating with the bladder was suspected. Exploratory laparotomy was performed, and the stone found in the ureter and removed by a longitudinal incision. It was 3 inches long and 1½ inch wide. The corresponding kidney was normal and its pelvis not dilated. The ureteral incision was united by a double row of interrupted fine silk sutures, not including the mucous membrane, and the peritoneum sutured over it. For two days there was slight hæmaturia, which had never before been observed. Recovery was rapid and without rise of temperature. Henry Morris has collected 29 cases of ureteral lithotomy, four of which were performed through peritoneal incisions. Diagnosis is easier when the stone is impacted near the vesical orifice.

[3] Zentralb. f. Chir., April 25, 1903, p. 457. [4] Med. Rec., March 7, 1903, p. 398. [5] Lancet, June 6, 1903, p. 1587. [6] Vide Abst. Zentralb. f. Chir, May 9, 1903, p. 509. [7] Vide Abst. Brit. Med. Jour., March 21, 1903, p. 691.

